# Supplementary material for: Improving the accuracy and precision of disease identification when utilizing EHR data for research: the case for hepatocellular carcinoma
Source: BMC Res Notes. 2025 Oct 1;18:410. doi: 10.1186/s13104-025-07465-z (PMC12487526; doi:10.1186/s13104-025-07465-z)
Supplement: Supplementary file 1 — Supplementary Material 1. [file 13104_2025_7465_MOESM1_ESM.docx]

**Online** **Supplementary Material**

**Table 1. ICD Codes to Identify Patients with Chronic Liver Disease**

| **Chronic Liver Disease** | **ICD Code** | **Description** |
| --- | --- | --- |
| Alcohol-associated liver disease | 571.0  571.1  571.2  571.3  K70.0  K70.10  K70.11  K70.2  K70.30  K70.31  K70.40  K70.41  K70.9 | Alcoholic fatty liver  Acute alcoholic hepatitis  Alcoholic cirrhosis of liver  Alcoholic liver damage, unspecified  Alcoholic fatty liver  Alcoholic hepatitis without ascites  Alcoholic hepatitis with ascites  Alcoholic fibrosis and sclerosis of liver  Alcoholic cirrhosis of liver without ascites  Alcoholic cirrhosis of liver with ascites  Alcoholic hepatic failure without coma  Alcoholic hepatic failure with coma  Alcoholic liver disease, unspecified |
| Metabolic dysfunction-associated steatotic liver disease | 571.8  571.9  K75.81  K76.0 | Other chronic nonalcoholic liver disease  Unspecified chronic liver disease without mention of alcohol  Nonalcoholic steatohepatitis  Fatty change of the liver, not elsewhere classified |
| Hepatitis B | 070.2  070.20  070.22  070.23  070.3  070.30  070.31  070.32  070.33  V02.61  B19.11  B16.9  B18.0  B18.1  B19.10  Z22.51 | Viral hepatitis B with hepatic coma  Viral hepatitis B with hepatic coma, acute or unspecified, without mention of hepatitis delta  Chronic hepatitis B with hepatic coma without hepatitis delta  Chronic viral hepatitis B with hepatic coma with hepatitis delta  Viral hepatitis B without mention of hepatic coma  Viral hepatitis B without mention of hepatic coma, acute or unspecified, without mention of hepatitis delta  Viral hepatitis B without mention of hepatic coma, acute or unspecified, with hepatitis delta  Chronic viral hepatitis B without mention of hepatic coma without mention of hepatitis delta  Chronic viral hepatitis B without mention of hepatic coma with hepatitis delta  Hepatitis B carrier  Unspecified viral hepatitis B with hepatic coma  Acute hepatitis B without hepatitis delta and without hepatic coma  Chronic viral hepatitis B with hepatitis delta  Chronic viral hepatitis B without hepatitis delta  Unspecified viral hepatitis B without hepatic coma  Carrier of viral hepatitis B |
| Hepatitis C | 070.44  070.54  070.7  070.70  070.71  B18.2  B19.20  B19.21 | Chronic hepatitis C with hepatic coma  Chronic hepatitis C without mention of hepatic coma  Unspecified viral hepatitis C  Unspecified viral hepatitis C without hepatic coma  Unspecified viral hepatitis C with hepatic coma  Chronic viral hepatitis C  Unspecified viral hepatitis C without hepatic coma  Unspecified viral hepatitis C with hepatic coma |
| Other viral hepatitis | B18.8  B18.9  B19.9  K73.0  K73.1  K73.2  K73.8  K73.9  K75.2 | Other chronic viral hepatitis  Chronic viral hepatitis, unspecified  Unspecified viral hepatitis without hepatic coma  Chronic persistent hepatitis, not elsewhere classified  Chronic lobular hepatitis, not elsewhere classified  Chronic active hepatitis, not elsewhere classified  Other chronic hepatitis, not elsewhere classified  Chronic hepatitis, unspecified  Nonspecific reactive hepatitis |
| Other chronic liver disease | 273.4  E88.01  571.42  K75.4  275.01  E83.110  E83.119  275.1  E83.01 | Alpha-1-antitrypsin deficiency  Alpha-1-antitrypsin deficiency  Autoimmune hepatitis  Autoimmune hepatitis  Hereditary hemochromatosis  Hereditary hemochromatosis  Hemochromatosis, unspecified  Disorders of copper metabolism  Wilson disease |
| Biliary cirrhosis | 571.6  K74.3  K74.4  K74.5 | Biliary cirrhosis  Primary biliary cirrhosis  Secondary biliary cirrhosis  Biliary cirrhosis, unspecified |
| Cirrhosis | 456.0  456.1  456.20  456.21  567.23  572.2  572.3  572.4  572.8  573.5  I85.00  I85.01  I85.10  I85.11  K65.2  K71.7  K72.90  K72.91  K74.0  K74.2  K74.60  K76.6  K76.7  K76.81  P78.81 | Esophageal varices with bleeding  Esophageal varices without mention of bleeding  Esophageal varices in diseases classified elsewhere, with bleeding  Secondary esophageal varices without bleeding  Spontaneous bacterial peritonitis  Hepatic encephalopathy  Portal hypertension  Hepatorenal syndrome  Other sequelae of chronic liver disease  Hepatopulmonary syndrome  Esophageal varices without bleeding  Esophageal varices with bleeding  Secondary esophageal varices without bleeding  Secondary esophageal varices with bleeding  Spontaneous bacterial peritonitis  Toxic liver disease with fibrosis and cirrhosis of liver  Unspecified hepatic failure  Hepatic failure, unspecified with coma  Hepatic fibrosis  Hepatic fibrosis with hepatic sclerosis  Unspecified cirrhosis of the liver  Portal hypertension  Hepatorenal syndrome  Hepatopulmonary syndrome  Congenital cirrhosis |
| Cryptogenic cirrhosis | 571.5  K74.69 | Cirrhosis of liver, not otherwise specified  Other cirrhosis of the liver |
| Hepatocellular carcinoma | 155  C22.0 | Malignant neoplasm of the liver and intrahepatic bile ducts  Liver cell carcinoma |
| Other liver or biliary cancer | 155.1  155.2  C22.1  C22.2  C22.3  C22.4  C22.7  C22.8  C22.9  C78.7  Z85.05 | Malignant neoplasm of intrahepatic bile ducts  Malignant neoplasm of liver, not specified as primary or secondary  Intrahepatic bile duct carcinoma  Hepatoblastoma  Angiosarcoma of the liver  Other sarcomas of the liver  Other specified carcinomas of liver  Malignant neoplasm of liver, primary, unspecified as to type  Malignant neoplasm of liver, not specified as primary or secondary  Secondary malignant neoplasm of liver and intrahepatic bile ducts  Personal history of malignant neoplasm or the liver or liver cancer |
